# The mediating role of emotional intelligence in the relationship between technostress and burnout prevention among critical care nurses a structural equation modelling approach

**DOI:** 10.1186/s12912-025-02852-0

**Published:** 2025-03-06

**Authors:** Mostafa Shaban, Mohamed Ezzelregal Abdelgawad, Shimmaa Mohamed Elsayed, Haitham Mokhtar Mohamed Abdallah

**Affiliations:** 1 Geriatric Nursing - Faculty of Nursing, Cairo University, Cairo, Egypt; 2 Critical care and emergency nursing, Faculty of Nursing, Alexandria University, Alexandria, Egypt; 3 Assistant Professor, Medical Surgical Nursing Department, College of Nursing, Jouf University, Sakaka, Al Jouf, Saudi Arabia; 4 Critical care and emergency department, Faculty of nursing, Damanhur university, Damanhur, Egypt

**Keywords:** Technostress, Burnout, Emotional intelligence, Critical care nurses, Structural equation modeling

## Abstract

**Background:**

Critical care nurses frequently experience high levels of technostress due to the increasing demands of healthcare technology, which contributes to burnout. Emotional intelligence has been shown to buffer stress in demanding environments, potentially mitigating burnout. However, its mediating role in the relationship between technostress and burnout among critical care nurses remains underexplored.

**Aim:**

This study aims to examine the mediating role of emotional intelligence in the relationship between technostress and burnout among critical care nurses.

**Methods:**

A cross-sectional study was conducted among 180 critical care nurses from two hospitals in Damnhour City, Egypt. Data were collected using the Technostress Questionnaire, Copenhagen Burnout Inventory (CBI), and Emotional Intelligence Scale. Structural Equation Modeling (SEM) was used to test the hypothesized relationships between technostress, emotional intelligence, and burnout, with bootstrapping employed to assess mediation.

**Results:**

Technostress was positively correlated with burnout (*r* = 0.56, *p* < 0.01), while emotional intelligence was negatively correlated with both technostress (*r* = -0.45, *p* < 0.01) and burnout (*r* = -0.49, *p* < 0.01). SEM analysis revealed that emotional intelligence significantly mediated the relationship between technostress and burnout (indirect effect = 0.23, *p* = 0.002), reducing burnout levels.

**Conclusion:**

Emotional intelligence plays a crucial role in mitigating the effects of technostress on burnout among critical care nurses. Targeted interventions to enhance emotional intelligence may help reduce burnout in technology-driven healthcare environments.

**Supplementary Information:**

The online version contains supplementary material available at 10.1186/s12912-025-02852-0.

## Introduction

The critical care nurses continuously encounter the highly pressurized and stressful environment within the fast-changing atmosphere of healthcare [1]. Many roles within the area involve dealing with life-threatening situations, making profound decisions, and even attending to emotional and physical needs on the part of the patients within the intensive care units [2, 3]. Added to that is the increased dependency on technology for the contemporary healthcare systems. While technological changes have revolutionized how health services are delivered, they have also come along with their demands and extra stressors coupled as technostress [4]. Technostress is a condition upon which a person feels unable to cope with new technologies and subsequently feels frustrated, anxious, and emotionally exhausted [5]. For critical care nurses, who encompass both technical and emotional complexities in their work, the experience of technostress is most poignant and might lead to burnout that affects their well-being and job performance [6, 7].

Burnout in health services is an omnipresent problem and could be defined as a syndrome characterized by emotional exhaustion, depersonalization, and decrease in personal accomplishment [8]. Critical care nurses, due to the nature of their work, hold a high disposition for burnout. The constant exposure to critically ill patients, less-than-predictable work environments, and emotional strain can take a significant toll on their mental and physical health [9]. Burnout affects not only the individual nurse but also patient care, healthcare organizations, and the broader healthcare system [10]. Burned-out nurses may become less concerned with their work, which can translate into lower levels of patient satisfaction, more medical errors, and higher levels of nurse turnover [11]. It follows, then, that addressing the factors contributing to burnout in critical care nursing is crucial not only for improving a nurse’s well-being but also for ensuring better patient outcomes [12, 13].

The association between emotional intelligence and burnout has already been considered in many contexts; however, possible mediation by emotional intelligence in the context of technostress has been much less well considered to date [14]. With the continuous development of health care technologies, a critical care nurse is expected to refresh his or her technical skills continuously, work with complicated systems, and incorporate new technologies into patient care [15]. For some nurses, this adaptation to technology may promote feelings of inadequacy, frustration, and even fear of becoming obsolete-some components of technostress [16]. It is still possible that emotional intelligence buffers these negative effects by providing nurses with the emotional means of managing technology use-related stress [17]. For instance, nurses with high emotional intelligence might manage their anxiety about learning new technologies more effectively, seek more support from colleagues, and also maintain a more consistently positive attitude toward change [18, 19].

The interplay of technostress, emotional intelligence, and burnout finds special significance in critical care nursing, which earnestly requires both emotional and technical competencies [19]. Critical care nurses need to cope not only with the emotional demands of their work but also to maintain their proficiency in the use of a wide scope of medical technologies, ranging from ventilators to electronic health records and telemedicine systems [20, 21]. The perpetual need to adapt to new technological tools may create a feeling of being overwhelmed that culminates in technostress [22]. If left unaddressed, this technostress contributes to burnout, further increasing the issues that critical care nurses have to face [22]. On the other hand, emotional intelligence could play a critical buffer in that relationship by helping nurses manage the emotional demands associated with technostress and preventing burnout [23].

As the healthcare system continues to progress into new dimensions, the compulsion to deal with the effects of technostress among critical care nurses has never been higher than now [24]. Emotional intelligence is one of the promising avenues that may help mitigate the adverse effects of technostress and prevent burnout [25]. This emerging literature underlines that emotional intelligence in healthcare settings may become of great importance and utilizes fully the potential to address challenges associated with technostress and burnout amongst critical care nurses [26]. By fostering emotional intelligence within the setting of the health care environment, one is able to help both in enhancing the well-being of the nurses as well as providing good quality of care to the patients [27].

### Aim of the study

This study aims to examine the mediating role of emotional intelligence in the relationship between technostress and burnout among critical care nurses. Specifically, it seeks to determine whether emotional intelligence serves as an intermediary mechanism through which technostress influences burnout, thereby offering insights into potential strategies for mitigating burnout in technology-intensive healthcare environments.

### Hypothesis

- H1: There is a positive association between technostress and burnout among critical care nurses.
- H2: Emotional intelligence is negatively related to burnout among critical care nurses.
- H3: Emotional intelligence mediates the relationship between technostress and burnout among critical care nurses, such that higher emotional intelligence partially explains the indirect pathway through which technostress contributes to burnout.

## Method

### Design

This study utilized a cross-sectional, correlational design to examine the relationships between technostress, emotional intelligence, and burnout among critical care nurses. Structural Equation Modelling (SEM) was employed to test the mediating role of emotional intelligence, as SEM is well-suited for evaluating complex relationships between multiple variables while accounting for measurement error and indirect effects.

### Setting

The study was conducted in Damnhour City, which is in Behera Governorate, Egypt. Specifically, the study was done in two major hospitals that currently have fully operating Intensive Care Units (ICUs). The advanced technological infrastructure and the critical nature of patient care made these hospitals ideal settings to study technostress and burnout among critical care nurses. Nurses working in the ICU environment face high- stakes situations with critical patients who often need various medical technologies, such as ventilators, electronic health records, and telemedicine systems. In these settings, the constant technological demands drive technostress; thus, they provide an ideal setting where the effect of emotional intelligence on mitigating burnout can be explored.

### Sample and sampling

The target population for this present study included the registered critical care nurses who were working in the Intensive Care Units (ICUs) of two major hospitals in Damnhour City, Behera, Egypt. Critical care nurses were selected because of the nature of their work, which entailed high emotional demands and extensive use of healthcare technologies, making them particularly vulnerable to technostress and burnout. The study sought to examine how emotional intelligence may mitigate technostress in this high-stress group.

### Calculation of sample size

The study population consisted of all registered critical care nurses working in the Intensive Care Units (ICUs) of two hospitals in Damnhour City, Egypt. The total number of ICU nurses across both hospitals was approximately 450, forming the study’s population.

The required sample size was calculated using G*Power software, based on an anticipated effect size of 0.3, a statistical power of 0.80, and a significance level of 0.05. Following Kline’s (2015) recommendation that SEM studies should have a minimum of 200 participants for robust model estimation, we targeted a final sample size of 200 nurses. After accounting for non-responses and exclusions, 180 nurses participated in the study.

### Sampling method

A stratified random sampling approach was applied to ensure a representative sample across ICU specialties. Nurses were categorized based on years of experience (1–5 years, 6–10 years, and > 10 years) and ICU specialty (general ICU, cardiovascular ICU, neonatal ICU, and other specialized ICUs). Proportional random selection was conducted within each stratum.

Inclusion and Exclusion Criteria.

- Inclusion Criteria:
  - Registered nurses with a valid nursing license.
  - Employed in an ICU setting for at least one year to ensure sufficient exposure to ICU-related technostress and burnout factors.
  - Directly involved in patient care.
  - Provided informed consent.
- Exclusion Criteria:
  - Nurses in administrative or non-clinical roles, as they may not experience the same technostress and burnout levels.
  - Nurses on extended leave (e.g., maternity, medical, or sabbatical leave) during the study period.
  - Nurses who declined participation in the study.

#### Clarification of exclusion criteria issue

Since the inclusion criterion required nurses to have at least one year of ICU experience, the exclusion of nurses with less than one year of experience was not necessary as a separate criterion. This has been corrected to avoid redundancy.

### Data collection instruments

To gather relevant data for the study, three well-established and validated instruments were employed: the Technostress Questionnaire, the Copenhagen Burnout Inventory (CBI), and the Emotional Intelligence Scale. These instruments were chosen for their widespread use in healthcare research and their proven reliability in assessing technostress, burnout, and emotional intelligence, respectively. Each tool was adapted for use in the local context, ensuring cultural relevance and linguistic accuracy through a translation and back-translation process. The instruments were administered in Arabic, the primary language of the participants.

#### Technostress questionnaire

To assess the level of technostress experienced by critical care nurses due to their interactions with technology, the authors developed a structured Technostress Questionnaire. This tool was specifically designed to capture key dimensions of technostress in the context of critical care nursing, considering the increasing demands of digital health technologies, electronic health records, telemedicine, and other digital interfaces in intensive care units (Appendix 1).

This questionnaire consisted of 20 items designed to assess five key dimensions of technostress:

- **Techno-overload**: The feeling that technology increases workload or requires a faster pace of work.
- **Techno-complexity**: The perception that technology is difficult to understand or too complex, leading to feelings of inadequacy.
- **Techno-insecurity**: Anxiety stemming from fear of job loss or obsolescence due to rapid technological changes.
- **Techno-uncertainty**: Stress related to constant technological updates or changes that make it difficult for nurses to maintain competence.
- **Techno-invasion**: The sense that technology intrudes into personal or professional boundaries, such as work-life balance disruptions.

Each item was rated on a 5-point Likert scale, ranging from 1 (strongly disagree) to 5 (strongly agree). Higher scores reflected greater levels of technostress. The overall technostress score was calculated by summing the scores across all items, with subscale scores calculated for each dimension. The Technostress Questionnaire has demonstrated high internal consistency in previous studies, with a Cronbach’s alpha of 0.85. In this study, the translated version also achieved a high reliability score (Cronbach’s alpha = 0.83).

#### Copenhagen burnout inventory (CBI)

The Copenhagen Burnout Inventory (CBI) is a widely recognized tool designed to measure burnout in professional and occupational settings [28]. Unlike other burnout assessment tools, the CBI focuses on three specific dimensions that encompass the multifaceted nature of burnout:

- **Personal burnout**: This dimension evaluates the degree of physical and emotional exhaustion experienced by an individual, regardless of their occupational role. It reflects general fatigue and exhaustion.
- **Work-related burnout**: This measures the extent to which work contributes to the feelings of exhaustion and stress. It specifically focuses on the demands and pressures that stem from the occupational environment.
- **Client-related burnout**: This assesses the fatigue and emotional drain associated with working closely with clients or patients. It highlights the stress arising from interpersonal interactions and caregiving responsibilities.

The CBI is composed of 19 items, rated on a 5-point Likert scale ranging from 1 (“never/almost never”) to 5 (“always”). Higher scores indicate higher levels of burnout in the respective dimensions. The instrument is valued for its clarity and adaptability across diverse work environments, particularly in healthcare settings where the emotional and physical demands are significant.

The CBI has been validated in numerous studies, demonstrating strong reliability and construct validity. In this study, it was culturally adapted for the target population and achieved a high Cronbach’s alpha score, indicating strong internal consistency, and client-related burnout (Cronbach’s α = 0.86).

#### Emotional intelligence scale

Emotional intelligence was assessed using the Wong and Law Emotional Intelligence Scale (WLEIS) [29], which was chosen for its applicability to workplace settings, particularly in healthcare. Emotional intelligence (EI) refers to the ability to perceive, understand, manage, and regulate emotions in oneself and others, which is crucial for managing stress and preventing burnout in high-pressure environments like critical care units.

The WLEIS consists of 33 items, divided into four subdomains:

1. Self-awareness: The ability to recognize and understand one’s own emotions.
2. Self-regulation: The ability to control or redirect disruptive emotions and impulses.
3. Empathy: The ability to recognize and understand the emotions of others.
4. Social skills: The ability to manage relationships and navigate social complexities effectively.

Each item was rated on a 5-point Likert scale, ranging from 1 (strongly disagree) to 5 (strongly agree), with higher scores reflecting greater emotional intelligence. Example items include statements such as “I have a good sense of why I have certain feelings most of the time” and “I am able to control my temper and handle difficulties rationally.”

The WLEIS has demonstrated high reliability in previous research, with Cronbach’s alpha values typically exceeding 0.80 for the total scale. In the present study, the translated and culturally adapted version of the scale was also pilot-tested to ensure it resonated well with Arabic-speaking ICU nurses. The reliability of the scale was confirmed with an overall Cronbach’s alpha of 0.88, demonstrating strong internal consistency across all four subdomains.

### Translation and pilot testing

All instruments were translated into Arabic following a translation-back-translation process. Initially, professional translators with expertise in both nursing and psychology translated the questionnaires from English to Arabic. The translated versions were then back-translated into English by independent bilingual experts to check for conceptual equivalence. Discrepancies between the original and back-translated versions were resolved through discussion between the translators and the research team to ensure both linguistic accuracy and cultural appropriateness.

A pilot study was conducted with 20 ICU nurses who were not part of the main study sample. These nurses completed the translated versions of all instruments, and their feedback was solicited regarding the clarity and relevance of each item. Minor revisions were made based on their input to improve the clarity of some items. Reliability testing using Cronbach’s alpha was also conducted during the pilot study, and all instruments demonstrated satisfactory reliability, with Cronbach’s alpha values ranging from 0.78 to 0.88.

### Scoring and interpretation

For each instrument, scoring was done according to the guidelines provided by the original developers. Total scores for technostress, emotional intelligence, and burnout were calculated by summing the individual item scores. Subscale scores were also computed for each dimension within the Technostress Questionnaire, CBI, and Emotional Intelligence Scale, providing a nuanced understanding of how each construct operates in the critical care nursing context.

Higher scores on the Technostress Questionnaire indicated higher levels of technostress, while higher scores on the CBI’s Emotional Exhaustion and Depersonalization subscales indicated greater burnout. In contrast, higher scores on the Personal Accomplishment subscale suggested lower burnout. For the Emotional Intelligence Scale, higher scores reflected higher emotional intelligence across all four subdomains.

### Data collection procedure

Data collection for the study was carried out over a six-week period, from September 1, 2024, to October 15, 2024, in two hospitals with Intensive Care Units (ICUs) located in Damnhour City, Behera, Egypt. A structured and systematic approach was followed to ensure high participation rates and reliable data collection, all while minimizing disruption to the nurses’ work schedules.

### Participant recruitment

Participants were recruited using a stratified random sampling method to ensure representation across various ICU departments (e.g., general ICU, neonatal ICU, cardiovascular ICU) and to capture a range of work experience levels. Nurses eligible to participate in the study were approached individually during their shift breaks or immediately after completing their shifts to minimize interference with their duties and to ensure they had sufficient time to consider their participation.

Nurses were given an informational sheet that detailed the purpose of the study, the voluntary nature of participation, and assurances regarding confidentiality. The research assistants explained that participation was entirely voluntary, that refusal to participate would not have any repercussions, and that they could withdraw at any stage of the study without any penalty. Written informed consent was obtained from each nurse before they participated in the study.

### Distribution and completion of questionnaires

Data collection was performed by a team of trained research assistants who were familiar with the study instruments and the ethical considerations involved in working with healthcare professionals. To minimize the risk of bias, the research assistants were not employed by the hospitals where the study took place and were instructed to remain neutral and not influence participants’ responses.

Nurses who consented to participate were provided with a packet containing the three study instruments: the Technostress Questionnaire, the Copenhagen Burnout Inventory (CBI), and the Emotional Intelligence Scale. The participants were asked to complete the questionnaires in a designated quiet area within the hospital, such as a break room or staff lounge, to ensure privacy and minimize distractions. On average, the completion of the questionnaires took approximately 20–30 min.

Participants were instructed to complete the questionnaires independently to ensure that their responses reflected their personal experiences and perceptions. Although research assistants were available to clarify any questions related to the meaning of the items, they were trained to refrain from providing any interpretation that might influence responses.

To further encourage participation and ensure that responses were as comprehensive as possible, participants who expressed concerns about completing the forms during their shifts were given the option to take the questionnaires home and return them in sealed envelopes at a later date. Envelopes were placed in secure drop boxes located in each ICU unit for convenient, anonymous return. These drop boxes were emptied daily by the research team to collect completed questionnaires and minimize the risk of lost or misplaced forms.

### Follow-up and maximizing participation

To maximize the response rate and ensure adequate sample size, follow-up reminders were conducted. Participants who had not completed or returned their questionnaires after two weeks were reminded verbally by the research assistants. A second reminder was provided during the third and fourth weeks of the data collection period. The reminders were conducted respectfully and in a manner that did not pressure or coerce the nurses, emphasizing the importance of their participation for the study’s success while reiterating the voluntary nature of the study.

Efforts were also made to schedule data collection during times that were convenient for the nurses. Data collection took place at different times of day to accommodate nurses working different shifts, including morning, evening, and night shifts. This flexible scheduling helped ensure that all eligible participants, regardless of their shift patterns, had the opportunity to participate in the study.

### Data analysis

Data were analyzed using the Statistical Package for the Social Sciences (SPSS) version 26 for descriptive statistics and AMOS for Structural Equation Modeling (SEM). Descriptive statistics were used to summarize the demographic characteristics of the sample, including age, gender, years of experience, and department. Means and standard deviations were calculated for the scores on the Technostress Questionnaire, CBI, and Emotional Intelligence Scale.

For inferential statistics, Pearson correlation analysis was conducted to examine the bivariate relationships between technostress, emotional intelligence, and burnout. Structural Equation Modeling (SEM) was then used to test the hypothesized mediating role of emotional intelligence in the relationship between technostress and burnout. Fit indices, including the chi-square statistic, Comparative Fit Index (CFI), and Root Mean Square Error of Approximation (RMSEA), were used to assess model fit. A *p*-value of < 0.05 was considered statistically significant. Before conducting Pearson correlation analysis, the normality of the dataset was evaluated using Shapiro-Wilk tests and Q-Q plots. The results confirmed that the data met the assumptions of normality, allowing for the appropriate use of Pearson correlation coefficients to examine relationships between the study variables.

The mediation effect was tested using bootstrapping methods, with 5,000 bootstrap samples and 95% confidence intervals to determine the indirect effects of emotional intelligence on the relationship between technostress and burnout. Both direct and indirect pathways were examined in the model.

### Ethical consideration

Ethical approval for the study was obtained from the Institutional Review Board (IRB) of the Faculty of Nursing at Damnhour University on August 21, 2022 (Research code: 44). All participants were informed of the study’s objectives, potential risks, and benefits prior to participation, and written informed consent was obtained from each participant. Confidentiality and anonymity were strictly maintained throughout the study. Data were de-identified and stored securely, accessible only to the research team. Participants were assured that their participation was voluntary and that they could withdraw from the study at any point without any consequences to their employment or professional standing.

The study adhered to the ethical principles outlined in the Declaration of Helsinki, ensuring that all participants were treated with respect and that their rights were protected throughout the research process.

## Results

Table 1 provides an overview of the demographic characteristics of the study participants (*N* = 180). The sample was predominantly female (67.8%), with 122 female participants compared to 58 males (32.2%). The majority of the participants were between the ages of 31 and 40 years (42.8%), followed by 36.1% in the 20–30 age group. Only 4.4% of participants were over 50 years of age. Regarding years of experience, 41.7% of the participants had between 6 and 10 years of experience, while 27.8% had 1–5 years of experience, and 30.5% had more than 10 years of experience. This distribution suggests that the sample consisted of relatively experienced nurses, with a notable proportion working in ICUs for over a decade. The distribution of participants across different ICU departments shows that 35.6% of nurses worked in general ICUs, 28.9% in cardiovascular ICUs, 20.0% in neonatal ICUs, and 15.6% in other ICU specialties.

**Table 1:** Demographic characteristics of participants (*N* = 180)

Variable	*n*	%
**Gender**		
Male	58	32.2
Female	122	67.8
**Age (years)**		
20–30	65	36.1
31–40	77	42.8
41–50	30	16.7
> 50	8	4.4
**Years of Experience**		
1–5	50	27.8
6–10	75	41.7
> 10	55	30.5
**Department**		
General ICU	64	35.6
Cardiovascular ICU	52	28.9
Neonatal ICU	36	20.0
Other (e.g., Surgical ICU)	28	15.6

Table 2 presents the descriptive statistics for technostress, emotional intelligence, and burnout among 180 critical care nurses. The average technostress score is 3.42, with a standard deviation of 0.78, indicating a moderate level of technostress on a scale from 1.2 to 5.0. Emotional intelligence scores are slightly higher, averaging 3.78 with a standard deviation of 0.85, suggesting that nurses generally possess moderate to high emotional intelligence within a range of 1.5 to 5.0. The total burnout score among the nurses’ averages at 4.05, with a standard deviation of 0.96 and a range from 2.1 to 6.0, pointing to moderate levels of overall burnout. This total includes personal burnout, which is slightly higher at 4.22, indicating significant emotional and physical exhaustion. Work-related burnout averages lower at 3.90, while client-related burnout is at 4.10, both reflecting substantial challenges but varying slightly in their impact on the nurses.

**Table 2:** Descriptive statistics for Technostress, Emotional Intelligence, and Burnout scores (*N* = 180)

Variable	Mean	SD	Range
**Technostress**	3.42	0.78	1.2–5.0
**Emotional Intelligence**	3.78	0.85	1.5–5.0
**Burnout (Total Score)**	4.05	0.96	2.1–6.0
- Personal burnout	4.22	0.91	2.0–6.0
- Work-related burnout:	3.90	0.84	1.5–5.8
- Client-related burnout	4.10	0.95	2.0–6.0

Table 3 presents the Pearson correlation coefficients between technostress, emotional intelligence, and the different dimensions of burnout. There was a significant positive correlation between technostress and burnout (*r* = 0.56, *p* < 0.01), indicating that higher levels of technostress were associated with higher burnout levels. In contrast, emotional intelligence was negatively correlated with burnout (*r* = -0.49, *p* < 0.01), suggesting that higher emotional intelligence is associated with lower burnout levels. Emotional intelligence was also negatively correlated with technostress (*r* = -0.45, *p* < 0.01), implying that nurses with higher emotional intelligence experienced less technostress. Interestingly, technostress showed a particularly strong relationship with emotional exhaustion (*r* = 0.52, *p* < 0.01) and depersonalization (*r* = 0.47, *p* < 0.01), two key components of burnout. Emotional intelligence, on the other hand, was most strongly correlated with personal accomplishment (*r* = 0.48, *p* < 0.01) and emotional exhaustion (*r* = -0.53, *p* < 0.01).

**Table 3:** Pearson Correlation Coefficients between Technostress, Emotional Intelligence, and Burnout dimensions

Variable	Technostress	Emotional Intelligence	Burnout (Total)	Emotional Exhaustion	Depersonalization	Personal Accomplishment
**Technostress**	1	-0.45**	0.56**	0.52**	0.47**	-0.30*
**Emotional Intelligence**	-0.45**	1	-0.49**	-0.53**	-0.41**	0.48**
**Burnout (Total)**	0.56**	-0.49**	1	0.80**	0.67**	-0.60**
**Emotional Exhaustion**	0.52**	-0.53**	0.80**	1	0.61**	-0.50**
**Depersonalization**	0.47**	-0.41**	0.67**	0.61**	1	-0.43**
**Personal Accomplishment**	-0.30*	0.48**	-0.60**	-0.50**	-0.43**	1

Note: *p* < 0.01 (**) and *p* < 0.05 (*)

Table 4 details the fit indices from the Structural Equation Modeling (SEM) analysis. The chi-square value of 158.32 with 88 degrees of freedom was statistically significant, but the chi-square/df ratio was 1.80, which falls within the acceptable range of ≤ 3.0, indicating a good fit. The Comparative Fit Index (CFI) and Tucker-Lewis Index (TLI) were 0.92 and 0.90, respectively, both above the acceptable cut-off of 0.90, demonstrating a good fit. The Root Mean Square Error of Approximation (RMSEA) was 0.05, and the Standardized Root Mean Square Residual (SRMR) was 0.04, both of which meet the criteria for a well-fitting model. The Goodness of Fit Index (GFI) was 0.91, slightly above the acceptable threshold of 0.90, while the Adjusted Goodness of Fit Index (AGFI) was 0.88, which is marginal but still within an acceptable range. Finally, the Akaike Information Criterion (AIC) of 206.32 was used for model comparison and confirmed that the model was well-specified.

**Table 4:** Structural equation modeling (SEM) fit indices

Fit Index	Value	Recommended Cut-off	Interpretation
**Chi-square (χ²)**	158.32	*p* > 0.05 (non-significant)	Acceptable
**Degrees of Freedom (df)**	88	N/A	N/A
**Chi-square/df (Normed Chi-square)**	1.80	≤ 3.0	Good Fit
**Comparative Fit Index (CFI)**	0.92	≥ 0.90	Acceptable Fit
**Tucker-Lewis Index (TLI)**	0.90	≥ 0.90	Acceptable Fit
**Root Mean Square Error of Approximation (RMSEA)**	0.05	≤ 0.06	Good Fit
**Standardized Root Mean Square Residual (SRMR)**	0.04	≤ 0.08	Good Fit
**Goodness of Fit Index (GFI)**	0.91	≥ 0.90	Acceptable Fit
**Adjusted Goodness of Fit Index (AGFI)**	0.88	≥ 0.85	Marginal Fit
**Akaike Information Criterion (AIC)**	206.32	Lower values indicate better fit	Good Fit (for model comparison)

Table 5 presents the standardized path coefficients for both the direct and indirect effects in the SEM analysis. The direct effect of technostress on burnout was significant, with a standardized coefficient of 0.42 (*p* < 0.001), showing that higher technostress leads to higher burnout. The negative direct effect of technostress on emotional intelligence was also significant (β = -0.47, *p* < 0.001), indicating that technostress reduces emotional intelligence. The direct effect of emotional intelligence on burnout was negative and significant (β = -0.49, *p* < 0.001), suggesting that higher emotional intelligence reduces burnout. The indirect effect of technostress on burnout through emotional intelligence was also significant (β = 0.23, *p* = 0.002), confirming that emotional intelligence mediates the relationship between technostress and burnout.

**Table 5:** SEM path coefficients for Direct and Indirect effects

Path	Standardized Coefficient (β)	*p*-value
**Technostress** ⟶ **Burnout (Direct)**	0.42	< 0.001
**Technostress** ⟶ **Emotional Intelligence**	-0.47	< 0.001
**Emotional Intelligence** ⟶ **Burnout**	-0.49	< 0.001
**Technostress** ⟶ **Burnout (Indirect)**	0.23 (through EI)	0.002

Table 6 summarizes the results of the bootstrap analysis used to test the mediation effect of emotional intelligence. The standardized indirect effect of technostress on burnout through emotional intelligence was 0.23, with a 95% confidence interval of 0.11 to 0.35, and a *p*-value of 0.002. Since the confidence interval does not include zero, the mediation effect is statistically significant.

**Table 6:** Bootstrap Analysis for Mediation Effect of Emotional Intelligence (5,000 samples)

Indirect Effect (Technostress ⟶ Burnout via Emotional Intelligence)	Standardized Indirect Effect	95% Confidence Interval	*p*-value
**Bootstrap Estimate**	0.23	0.11–0.35	0.002

**Fig. 1:**
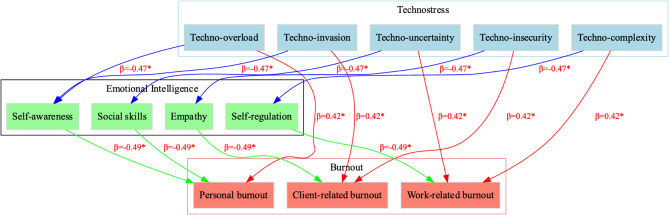
The path analysis model

Table 7 and Fig. 1 presents the mean burnout scores across different levels of emotional intelligence. Nurses with low emotional intelligence (EI ≤ 2.5) had the highest mean burnout score of 4.82 (SD = 0.89), whereas those with moderate emotional intelligence (EI between 2.6 and 3.9) had a lower mean burnout score of 4.12 (SD = 0.75). Nurses with high emotional intelligence (EI ≥ 4.0) reported the lowest burnout score, with a mean of 3.31 (SD = 0.68). The difference in burnout scores across these groups was statistically significant (*p* < 0.001).

**Table 7:** Burnout scores by Emotional Intelligence levels

Emotional Intelligence Level	*n*	Mean Burnout Score	SD	*p*-value
**Low (≤ 2.5)**	48	4.82	0.89	< 0.001
**Moderate (2.6–3.9)**	85	4.12	0.75	
**High (≥ 4.0)**	47	3.31	0.68	

## Discussion

This study examined the mediating role of emotional intelligence in the relationship between technostress and burnout among critical care nurses. The findings indicate that emotional intelligence explains part of the indirect pathway through which technostress contributes to burnout, rather than moderating its effects.

The positive correlation between technostress and burnout found in this study aligns with previous research, which consistently shows that increased exposure to technological demands contributes to higher levels of stress and emotional exhaustion among nurses [30]. In critical care settings, the frequent use of sophisticated medical technologies such as ventilators, electronic health records (EHRs), and diagnostic machines can lead to feelings of overload and frustration [31]. These demands, coupled with the pressure of delivering life-saving care, place critical care nurses in a unique position where they are particularly vulnerable to technostress [32]. Our findings highlight that technostress is significantly associated with burnout, particularly emotional exhaustion and depersonalization, two core dimensions of burnout that reflect a sense of fatigue and emotional detachment from the job.

The high levels of emotional exhaustion found among the participants echo the results of other studies that have documented similar trends in healthcare professionals working in high-stress environments [33]. Emotional exhaustion is often the first and most prominent symptom of burnout, and it is particularly concerning in nursing, where compassion, empathy, and patient-centered care are essential [34]. The link between technostress and emotional exhaustion in this study suggests that as technological demands increase, nurses may struggle to maintain the emotional energy required to care for critically ill patients [35]. Depersonalization, or the development of a cynical and detached attitude toward patients, is another burnout dimension strongly related to technostress [36]. This is particularly problematic in critical care settings, where patient-nurse interactions are often intense and emotionally charged [37]. As our study indicates, Nurses experiencing high levels of technostress may exhibit increased emotional exhaustion and reduced engagement in their work. Given that burnout can contribute to emotional detachment in clinical practice, addressing technostress could be essential in maintaining high-quality patient care [38].

Importantly, this study adds to the existing literature by highlighting the significant protective role of emotional intelligence in mitigating the effects of technostress on burnout [39]. Emotional intelligence, defined as the ability to perceive, understand, manage, and regulate emotions, has been shown to be a critical factor in helping individuals navigate stressful and emotionally demanding situations [40]. The negative association between emotional intelligence and burnout in this study suggests that nurses with higher levels of emotional intelligence are better equipped to manage the emotional challenges posed by technostress [41]. This is consistent with previous research indicating that emotionally intelligent individuals are more adept at managing their stress levels, maintaining interpersonal relationships, and preventing the onset of burnout [40, 42].

Our findings demonstrate that emotional intelligence significantly associated with emotional exhaustion and depersonalization. Nurses who possess high emotional intelligence are more likely to regulate their emotions effectively, avoid becoming overwhelmed by the emotional demands of the job, and maintain a sense of empathy and connection with their patients [43]. These nurses are less likely to experience emotional exhaustion because they can recognize the early signs of stress and take proactive measures to manage it [44]. Furthermore, emotional intelligence enables nurses to remain engaged and connected with their patients, reducing the likelihood of depersonalization, even in the face of significant stressors such as technostress [45].

The mediation analysis provides further evidence of the critical role of emotional intelligence in the relationship between technostress and burnout [46]. Specifically, emotional intelligence was found to partially mediate the impact of technostress on burnout, suggesting that while technostress directly contributes to burnout, The analysis indicates that emotional intelligence mediates the relationship between technostress and burnout, partially explaining how technostress contributes to burnout among critical care nurses [47]. This finding is important because it suggests that emotional intelligence not only acts as a buffer against burnout but also mitigates the negative effects of technostress, providing a pathway through which nurses can manage the demands of both technology and patient care more effectively.

The bootstrap analysis confirms the robustness of this mediation effect, with a significant indirect effect of technostress on burnout through emotional intelligence. These findings suggest that interventions aimed at enhancing emotional intelligence may help reduce burnout by addressing one of the pathways through which technostress contributes to it [48]. Emotional intelligence training programs could help nurses develop the skills necessary to recognize and manage the emotional and psychological challenges associated with technostress, thereby reducing their risk of burnout [49].

Our findings also align with previous research on the role of emotional intelligence in promoting resilience and psychological well-being in healthcare professionals [50]. Nurses with higher emotional intelligence are not only better at managing their own emotions but also at navigating the emotions of others, including their colleagues and patients [51]. This skill set is particularly important in critical care environments, where teamwork, communication, and emotional labor are essential components of effective patient care [52]. By fostering emotional intelligence, healthcare organizations may be able to enhance the emotional resilience of their nursing staff, leading to better job satisfaction, reduced turnover, and improved patient outcomes [53].

It is also worth noting that the association between emotional intelligence and personal accomplishment was positive, indicating that nurses with higher emotional intelligence are more likely to feel a sense of achievement and fulfillment in their work [54]. This finding underscores the broader benefits of emotional intelligence beyond just reducing stress and preventing burnout [55]. Nurses who are emotionally intelligent may find greater meaning and satisfaction in their work, which can contribute to a more positive workplace environment and higher levels of job engagement [56].

### Implications of the study

The findings of this study carry several important implications for nursing practice, healthcare management, and future research. First and foremost, the demonstrated mediating role of emotional intelligence in explaining the indirect relationship between technostress and burnout underscores the need for healthcare organizations to prioritize emotional intelligence development as part of ongoing professional training for nurses. Implementing emotional intelligence training programs in nursing curricula and continuing education initiatives could enhance nurses’ abilities to manage stress and emotional exhaustion, thereby reducing burnout rates.

Healthcare institutions should also consider integrating emotional intelligence assessments into hiring processes and performance evaluations to identify nurses who may be at greater risk of experiencing technostress and burnout. By doing so, organizations can provide targeted support and interventions for those individuals, creating a more resilient and emotionally equipped workforce. Furthermore, given the significant relationship between technostress and burnout, efforts to improve the design and usability of medical technologies, alongside proper training and technical support for nurses, are crucial in reducing technostress.

From a research perspective, this study opens the door for further exploration into the role of emotional intelligence in various healthcare settings beyond critical care nursing. Future studies can examine how emotional intelligence moderates other workplace stressors and investigate interventions that could foster emotional intelligence in high-stress environments. Additionally, this research provides a foundation for developing more comprehensive models that include other personal and environmental factors contributing to burnout and technostress.

### Limitations

This study has several limitations that should be acknowledged. First, the cross-sectional design of the study limits the ability to draw causal inferences. While the relationships between technostress, emotional intelligence, and burnout were significant, it is unclear whether technostress directly causes burnout or whether emotional intelligence serves as a mechanism through which technostress contributes to burnout over time. Longitudinal studies are needed to explore the directionality of these relationships and determine whether emotional intelligence interventions lead to sustained reductions in burnout.

Second, the study was conducted in a specific geographic location—Damnhour City, Behera, Egypt—and the sample was limited to critical care nurses in two hospitals. This may affect the generalizability of the findings to other healthcare contexts or nursing populations in different regions or countries. The unique cultural, organizational, and technological environments of the study setting may have influenced the results, and future research should seek to replicate these findings in more diverse settings to validate their broader applicability.

Another limitation relates to the use of self-reported measures for technostress, emotional intelligence, and burnout. Although the instruments used were well-validated, self-reported data may be subject to social desirability bias or inaccuracies due to participants’ perceptions of their own emotional intelligence and stress levels. Objective measures of emotional intelligence and technostress, along with qualitative data, could provide a more comprehensive understanding of these constructs in future research.

Finally, while the sample size of 180 participants was adequate for the analyses performed, a larger sample size could enhance the statistical power of the study and allow for more detailed subgroup analyses. Further research with larger and more varied samples could provide a deeper understanding of how demographic factors such as age, gender, and experience influence the relationships between technostress, emotional intelligence, and burnout.

## Conclusion

This study highlights the mediating role of emotional intelligence in the relationship between technostress and burnout, demonstrating how it helps explain the indirect pathway through which technostress contributes to burnout among critical care nurses. In a high-pressure environment where technological demands and emotional labor converge, emotional intelligence emerges as a protective factor that helps nurses manage the psychological and emotional toll of their work. The findings highlight that while technostress is a significant contributor to burnout, particularly in terms of emotional exhaustion and depersonalization, nurses with higher emotional intelligence are better equipped to buffer these effects and maintain their well-being.

As healthcare technology continues to evolve and play a central role in clinical practice, it is imperative for healthcare organizations to recognize the potential risks of technostress and take proactive steps to support their staff. This includes not only providing adequate training and resources for managing new technologies but also fostering emotional resilience through emotional intelligence development programs. By investing in emotional intelligence, healthcare organizations can reduce burnout, improve job satisfaction, and ultimately enhance the quality of care delivered to patients.

In conclusion, the findings of this study underscore the importance of addressing both the emotional and technological challenges faced by critical care nurses. Emotional intelligence represents a critical resource in navigating these challenges and promoting sustainable, healthy work environments in an increasingly technology-driven healthcare landscape.

## Electronic supplementary material

Below is the link to the electronic supplementary material.

Supplementary Material 1

## Data Availability

The datasets generated during and/or analyzed during the current study are available from the corresponding author on reasonable request.
