# Supplementary material for: The mediating role of emotional intelligence in the relationship between technostress and burnout prevention among critical care nurses a structural equation modelling approach
Source: BMC Nurs. 2025 Mar 6;24:255. doi: 10.1186/s12912-025-02852-0 (PMC11887162; doi:10.1186/s12912-025-02852-0)
Supplement: Supplementary file 1 — Supplementary Material 1 [file 12912_2025_2852_MOESM1_ESM.pdf]

Appendix: Technostress Questionnaire

(Developed by the Authors for the Current Study)

Instructions:

Please indicate how much you agree or disagree with each statement using the following scale:
1 = Strongly Disagree
2 = Disagree
3 = Neutral
4 = Agree
5 = Strongly Agree

Techno-Overload (Increased Workload Due to Technology)

1. 1. The use of technology in the ICU increases my overall workload.
2. 2. I feel pressured to complete tasks more quickly due to the use of digital systems.
3. 3. The amount of patient data and digital documentation I have to handle is overwhelming.
4. 4. I am required to multitask more frequently because of digital healthcare technologies.

Techno-Complexity (Difficulty in Understanding and Using Technology)

5. 1. Learning to use new healthcare technologies is difficult for me.
6. 2. I often feel that I lack the necessary technical skills to fully utilize digital systems.
7. 3. The complexity of medical technology makes my work more stressful.
8. 4. I feel frustrated when I have to use multiple electronic systems for patient care.

Techno-Insecurity (Fear of Job Replacement or Inadequacy)

9. 1. I worry that automation and artificial intelligence (AI) may replace my role in patient care.
10. 2. The increasing dependence on technology makes me feel less valuable as a nurse.
11. 3. I feel that younger nurses are more proficient with technology, making me feel at a disadvantage.
12. 4. I fear that if I do not quickly adapt to new technologies, I may be left behind.

Techno-Uncertainty (Frequent Changes in Technology and Lack of Training)

13. 1. Frequent updates and changes to hospital technologies disrupt my workflow.
14. 2. I find it stressful when new systems are introduced without proper training.
15. 3. The electronic health record (EHR) system is updated too frequently, making it difficult to keep up.
16. 4. Technical failures and system downtime increase my stress at work.

Techno-Invasion (Technology Intruding on Work-Life Balance and Patient Care)

17. 1. The use of technology makes it difficult for me to separate work from personal life.
18. 2. I feel that I am expected to be available outside of work due to digital communication systems.

19. 3. The increasing use of technology in patient care reduces my direct interaction with patients.
20. 4. I feel that technology dictates how I work, rather than supporting my nursing role.

Scoring and Interpretation:

• The total Technostress Score is obtained by summing all item scores.
• Higher scores indicate greater levels of technostress.
• Subscale scores can be calculated for each of the five dimensions.
